# Percutaneous dilatational tracheostomy: mostly safe, but do benefits outweigh risks?

**DOI:** 10.1186/cc13761

**Published:** 2014-03-11

**Authors:** Damon C Scales, Brian H Cuthbertson

**Affiliations:** 1 Department of Critical Care Medicine, Sunnybrook Health Science Centre, 2075 Bayview Avenue, Room D108, Toronto, Ontario M4N 3 M5, Canada; 2 Interdepartmental Division of Critical Care Medicine, University of Toronto, 2075 Bayview Avenue, Room D108, Toronto, Ontario M4N 3 M5, Canada; 3 Institute of Health Policy, Management and Evaluation, University of Toronto, Health Sciences Building, 155 College Street, Suite 425, Toronto, Ontario M5T 3 M6, Canada

## Abstract

Percutaneous dilatational tracheostomies have become one of the most frequently performed surgical procedures in the ICU, and are believed to offer a variety of advantages over open tracheostomies, including increased convenience. Recent publications have established that the risk of fatal complications related to the procedure is low. However, clinicians must still weigh these risks against expected but largely unproven benefits. More research is needed to establish the indications for the procedure, including the optimal patient selection and timing during a course of mechanical ventilation. Such studies should also seek to improve our ability to accurately identify which patients will require prolonged mechanical ventilation, and to quantify the potential benefits of tracheostomy compared with prolonged translaryngeal intubation.

## 

In a previous issue of *Critical Care*, Simon and colleagues provide a contribution that helps clarify the safety of the percutaneous dilatational tracheostomy procedure [1]. Tracheostomy is one of the most common surgical procedures performed in the ICU [2], and percutaneous tracheostomies have now largely replaced the open tracheostomy technique [3,4]. The percutaneous approach allows the procedure to be performed in the ICU instead of in the operating room, making it more convenient to arrange and avoiding competition for limited operating room resources [5]. The percutaneous approach is also a skill that can be taught to intensivists, whereas surgeons largely performed open tracheostomies.

Most evaluations of percutaneous tracheostomies have suggested that their safety profile is similar to that of the open procedure in unselected ICU patients [5]. However, there are several problems inherent to defining the safety of new procedures. For example, safety information derived from comptive randomised trials may fail to detect safety concerns because of limited sample size and restrictive inclusion and exclusion criteria. Adverse events that occur in routine practice are seldom captured by the medical literature, introducing a potential for publication and reporting bias [6]. Finally, the requirements for post-market surveillance that have been developed for pharmaceutical therapies seldom exist for new surgical techniques.

Simon and colleagues systematically reviewed the peer-reviewed literature spanning a 28-year period to catalogue and describe the complications reported to be associated with the procedure [1]. In addition, they went to great lengths (including contacting all corresponding authors) to estimate the denominator for these published reports, a necessary step for estimating the incidence of associated complications. To corroborate these risk estimates, they also augmented the literature review with a thorough analysis of their own departmental records representing real-world data and an accurate local estimate of complications relative to total number of procedures. They concluded that the incidence of lethal complications is low: only 1.7 deaths per 1,000 procedures. Furthermore, the greatest risk of fatality occurred during the procedure (nearly one-third of deaths), suggesting that there still may be opportunities to improve the safety of the operative technique and reduce complications [7]. Finally, this study from Simon and colleagues suggests several potential risk factors for encountering complications, although their methodology precluded a systematic evaluation of these factors.

Future research in this field should attempt to answer a number of remaining research questions. We need to identify the residual role for the open tracheostomy, especially since these procedures are becoming less common and are only being performed in the most technically challenging and highest risk patients. There is still a need for further research to inform the indications for all types of tracheostomy, including the optimal patient selection and timing during an episode of mechanical ventilation [8]. Although the risk of percutaneous tracheostomy may seem low, whether patients should be subjected to this risk without clear benefits to the procedure is debatable.

With the publication of the TracMan trial, we know that there is no benefit of early (≤4 days) versus late (≥10 days) tracheostomy [9]. Future studies should therefore focus on the benefits and risks of avoiding tracheostomy in favour of prolonged translaryngeal intubation, as well as on the value of late versus later tracheostomies in general ICU patients. We also need to improve our ability to accurately identify which patients will require prolonged mechanical ventilation both to aid clinical decision-making and to improve study validity [9,10]. Finally, we believe that some patient populations – for example, those with chronic respiratory conditions or underlying neurological injury – may have risk–benefit profiles that differ from general ICU patients, and this should be further explored.

### Competing interests

DCS holds a New Investigator Award from the Canadian Institutes for Health Research and a Fellowship in Translational Health Research from Physicians’ Services Incorporated Foundation. BHC declares that he has no competing interests.
